# ASO Author Reflections: First Descriptions of Patients with Pregnancy Associated Breast Cancer and Subsequent Pregnancy

**DOI:** 10.1245/s10434-024-15854-0

**Published:** 2024-07-23

**Authors:** Alissa Doll, Nimmi S. Kapoor

**Affiliations:** Department of Surgery, Division of Surgical Oncology, David Geffen School of Medicine, University of California Los Angeles, Los Angeles, CA USA

## Past

After treatment of pregnancy associated breast cancer (PABC), some women desire future pregnancy. As the maternal age rises and incidence of breast cancer diagnosed in young women increases,^1,2^ increasing numbers of young women consider their families to be incomplete after breast cancer treatment.^3^ While safety of pregnancy after breast cancer has been demonstrated through studies such as the POSTIVE trial,^4^ the same cannot necessarily be said about women with PABC. Evidence to guide these patients in decision-making is lacking.

## Present

In this retrospective review, we captured the incidence and clinical outcomes of patients with PABC with and without subsequent pregnancies after their breast cancer treatment between 2010 and 2023 from a single institution^5^. We defined PABC to include patients diagnosed with pregnancy during breast cancer and up to 5 years postpartum. Ultimately, our goal was to provide data to develop recommendations in the growing population of patients who desire pregnancy following PABC. Among 75 patients diagnosed with PABC, 17 patients had a documented subsequent pregnancy and 58 did not have a subsequent pregnancy. We found that women with subsequent pregnancy were younger and less likely to have had a prior pregnancy compared with those who did not have a subsequent pregnancy. Treatment between the two groups was similar with regards to neoadjuvant chemotherapy, adjuvant chemotherapy, breast surgery, axillary surgery, reconstructive surgery, adjuvant radiation, and adjuvant oral endocrine therapy. While we found no difference in disease-free survival, overall survival, or distant disease-free survival at 5 years between the two groups, our study size was small, and follow-up was limited. Of the patients with PABC with subsequent pregnancy, 14 of 17 patients had successful deliveries.

## Future

Young patients with PABC who have fewer children are more likely to desire future pregnancy. This study provides promising initial data supporting the safety of pursuing pregnancy after breast cancer treatment for patients with PABC. Future studies are needed to evaluate the use and safety of fertility treatment, surgical choices, and psychosocial challenges these younger patients may face. Long-term pooled analyses are needed to develop further guidelines to better counsel our patients on the risks and benefits of future pregnancies after PABC treatment.
